# Author Correction: Antiviral fibrils of self-assembled peptides with tunable compositions

**DOI:** 10.1038/s41467-024-46005-4

**Published:** 2024-02-19

**Authors:** Joseph Dodd-o, Abhishek Roy, Zain Siddiqui, Roya Jafari, Francesco Coppola, Santhamani Ramasamy, Afsal Kolloli, Dilip Kumar, Soni Kaundal, Boyang Zhao, Ranjeet Kumar, Alicia S. Robang, Jeffrey Li, Abdul-Rahman Azizogli, Varun Pai, Amanda Acevedo-Jake, Corey Heffernan, Alexandra Lucas, Andrew C. McShan, Anant K. Paravastu, B. V. Venkataram Prasad, Selvakumar Subbian, Petr Král, Vivek Kumar

**Affiliations:** 1 Department of Biomedical Engineering, New Jersey Institute of Technology, Newark, NJ 07102 USA; 2 Department of Chemistry, University of Illinois at Chicago, Chicago, IL 60607 USA; 3 Public Health Research Institute, New Jersey Medical School, Rutgers University, Newark, NJ 07103 USA; 4 Department of Molecular Virology & Microbiology, Baylor College of Medicine, Houston, TX 77030 USA; 5 School of Chemical and Biomolecular Engineering, Georgia Institute of Technology, Atlanta, GA 30332 USA; 6 Department of Biological Sciences, New Jersey Institute of Technology, Newark, NJ 07102 USA; 7 SAPHTx Inc, Newark, NJ 07104 USA; 8 Center for Personalized Diagnostics and Center for Immunotherapy Vaccines and Virotherapy, Biodesign Institute, Arizona State University, 727 E, Tempe, AZ USA; 9 School of Chemistry and Biochemistry, Georgia Institute of Technology, Atlanta, GA 30332 USA; 10 Department of Physics, University of Illinois at Chicago, Chicago, IL 60607 USA; 11 Department of Pharmaceutical Sciences, University of Illinois at Chicago, Chicago, IL 60607 USA; 12 Department of Chemical Engineering, University of Illinois at Chicago, Chicago, IL 60607 USA; 13 Department of Chemical and Materials Engineering, New Jersey Institute of Technology, Newark, NJ 07102 USA; 14 Department of Endodontics, Rutgers School of Dental Medicine, Newark, NJ 07103 USA

**Keywords:** Drug delivery, Protein design, Biomaterials - proteins

Correction to: *Nature Communications* 10.1038/s41467-024-45193-3, published online 07 February 2024

This Article contains an error in the spelling of the author Boyang Zhao, which was incorrectly given as Boyan Zhao. The error has been corrected in the PDF and HTML versions of the Article.

