# Author Correction: Antiviral fibrils of self-assembled peptides with tunable compositions

**DOI:** 10.1038/s41467-024-51775-y

**Published:** 2024-08-28

**Authors:** Joseph Dodd-o, Abhishek Roy, Zain Siddiqui, Roya Jafari, Francesco Coppola, Santhamani Ramasamy, Afsal Kolloli, Dilip Kumar, Soni Kaundal, Boyang Zhao, Ranjeet Kumar, Alicia S. Robang, Jeffrey Li, Abdul-Rahman Azizogli, Varun Pai, Amanda Acevedo-Jake, Corey Heffernan, Alexandra Lucas, Andrew C. McShan, Anant K. Paravastu, B. V. Venkataram Prasad, Selvakumar Subbian, Petr Král, Vivek Kumar

**Affiliations:** 1 Department of Biomedical Engineering, New Jersey Institute of Technology, Newark, NJ 07102 USA; 2 Department of Chemistry, University of Illinois at Chicago, Chicago, IL 60607 USA; 3 Public Health Research Institute, New Jersey Medical School, Rutgers University, Newark, NJ 07103 USA; 4 Department of Molecular Virology & Microbiology, Baylor College of Medicine, Houston, TX 77030 USA; 5 School of Chemical and Biomolecular Engineering, Georgia Institute of Technology, Atlanta, GA 30332 USA; 6 Department of Biological Sciences, New Jersey Institute of Technology, Newark, NJ 07102 USA; 7 SAPHTx Inc, Newark, NJ 07104 USA; 8 Center for Personalized Diagnostics and Center for Immunotherapy Vaccines and Virotherapy, Biodesign Institute, Arizona State University, 727 E Tempe, AZ USA; 9 School of Chemistry and Biochemistry, Georgia Institute of Technology, Atlanta, GA 30332 USA; 10 Department of Physics, University of Illinois at Chicago, Chicago, IL 60607 USA; 11 Department of Pharmaceutical Sciences, University of Illinois at Chicago, Chicago, IL 60607 USA; 12 Department of Chemical Engineering, University of Illinois at Chicago, Chicago, IL 60607 USA; 13 Department of Chemical and Materials Engineering, New Jersey Institute of Technology, Newark, NJ 07102 USA; 14 Department of Endodontics, Rutgers School of Dental Medicine, Newark, NJ 07103 USA

**Keywords:** Drug delivery, Protein design, Biomaterials - proteins

Correction to: *Nature Communications* 10.1038/s41467-024-45193-3, published online 07 February 2024

The original version of this Article contained an error in the Acknowledgements, which incorrectly read ‘V.K. acknowledges support from the NIH NEI R15 EY029504, NIH NIDCR R01DE031812, NIH NIAMS R21AR079708; and the Undergraduate Research and Innovation program at NJIT.’ The correct version replaces this sentence with ‘V.K. acknowledges support from the NIH NEI R15 EY029504, NIH NIAMS R21AR079708; and the Undergraduate Research and Innovation program at NJIT.’ This has been corrected in both the PDF and HTML versions of the Article.

