# Supplementary material for: Antiviral fibrils of self-assembled peptides with tunable compositions
Source: Nat Commun. 2024 Feb 7;15:1142. doi: 10.1038/s41467-024-45193-3 (PMC10850501; doi:10.1038/s41467-024-45193-3)
Supplement: Supplementary file 1 — Supplementary Information [file 41467_2024_45193_MOESM1_ESM.pdf]

Supplementary Information for:

Antiviral Fibrils of Self-assembled Peptides with Tunable Compositions

Joseph Dodd-o[1,*], Abhishek Roy[1,*], Zain Siddiqui[1,], Roya Jafari[2], Francesco Coppola[2], Santhamani Ramasamy[3], Afsal Kolloli[3], Dilip Kumar[4], Soni Kaundal[4], Boyan Zhao[4], Ranjeet Kumar[3], Alicia S. Robang[5], Jeffrey Li[5], Abdul-Rahman Azizogli[6], Varun Pai[6], Amanda Acevedo-Jake[1], Corey Heffernan[1,7], Alexandra Lucas[8], Andrew C. McShan[9], Anant K. Paravastu[5], BV Venkataram Prasad[4], Selvakumar Subbian[3], Petr Král[2,10,11,12,^], Vivek Kumar[1,6,7,13,14,^]

[1] Department of Biomedical Engineering, New Jersey Institute of Technology, Newark, NJ, 07102

[2] Department of Chemistry, University of Illinois at Chicago, Chicago, IL, 60607

[3] Public Health Research Institute, New Jersey Medical School, Rutgers University, Newark, NJ, 07103

[4] Department of Molecular Virology & Microbiology, Baylor College of Medicine, Houston, TX, 77030

[5] School of Chemical and Biomolecular Engineering, Georgia Institute of Technology, Atlanta, GA, 30332

[6] Department of Biological Sciences, New Jersey Institute of Technology, Newark, NJ, 07102

[7] SAPHTx Inc, Newark, NJ, 07104

[8] Center for Personalized Diagnostics and Center for Immunotherapy Vaccines and Virotherapy, Biodesign Institute, Arizona State University, 727 E Tyler St, Tempe, AZ

[9] School of Chemistry and Biochemistry, Georgia Institute of Technology, Atlanta, GA 30332

[10] Department of Physics, University of Illinois at Chicago, Chicago, IL, 60607

[11] Department of Pharmaceutical Sciences, University of Illinois at Chicago, Chicago, IL, 60607

[12] Department of Chemical Engineering, University of Illinois at Chicago, Chicago, IL, 60607

[13] Department of Chemical and Materials Engineering, New Jersey Institute of Technology, Newark, NJ, 07102

[14] Department of Endodontics, Rutgers School of Dental Medicine, Newark, NJ, 07103

*: These authors contributed equally
^: These authors jointly supervised this work

A

Name	SA domain	Linker	Targeting	Ref.
SBP2			SALEEQLKTFLDKFMHELEDLLYQLAL	(23)
ESBP2	ESLSLSLSLSLSLE	G	SALEEQLKTFLDKFMHELEDLLYQLAL	

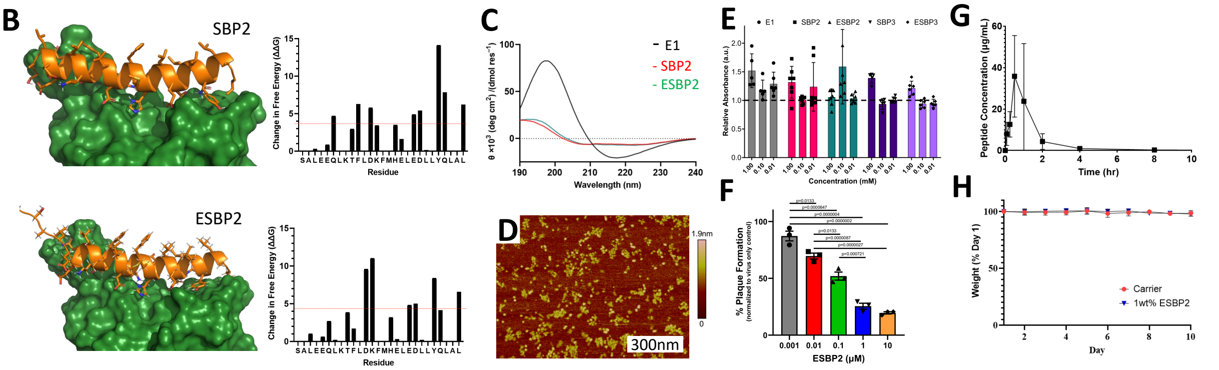

Supplementary Figure 1: Improvement of Spike targeting and inhibition of infection of non-human primate cells *in vitro*. **(A)** Mutation of SBP1 [23] to achieve a more stable α-helix and improved efficacy for SARS-CoV-2 yielded SBP2 (underlined bold residues are essential for spike binding and are conserved in all constructs). Appendage of the negative flanked SAP domain resulted in an aggregating construct with retained efficacy. **(B)** Comparative analysis of SBP2 and ESBP2 (both shown in orange) binding of Spike protein (shown in green) by *in silico* modeling. BUDE Alanine scanning identified specific residues contributing to the stability of SBP2 and ESBP2 binding of the spike protein/ACE-2 binding pocket. (Dotted red lines represent the threshold for identifying active binding residues). **(C)** CD confirmed the formation of β-sheet structures for E1 and α-helical structure for SBP2 and ESBP2. **(D)** AFM shows the formation of aggregates with few to no discernible fibrils assembled. (Scale bar: 300 nm).**(E)** The cytocompatibility of constructs was dose-dependently evaluated with Vero cells with peptides over 3 orders of magnitude (0.01-1 mM), establishing a wide range of *in vitro* safety for E1, SBP2, ESBP2, SBP3 and ESBP3 peptides. Mean ± SD, $p > 0.1$ for all groups. **(F)** ESBP2 showed significantly improved *in vitro* live virus inhibition compared to SBP2. (Mean ± SEM, n=3, ** $p<0.01$, * $p<0.05$). Data are presented as mean +/- SEM. One-way ANOVA (df=4, F-statistic=91.3, p=0.0000000791; Tukey Honestly Significant Difference (HSD) post-hoc test for multiple comparisons). **(G)** IV bolus administration of ESBP2 through the tail vein of Wistar rats (n=8) showed a concentration peak at 30 minutes and was eliminated by 8 hours. The observed maximum concentration was 36 µg/mL at 30 minutes following injection. The area under the curve was calculated to be 47 µg-hr/mL. **(H)** Daily repeated IV dosing with ESBP2 for 10 days showed no significant changes in body weight compared to the carrier (dose day 1). Source data are provided in the Source Data file.

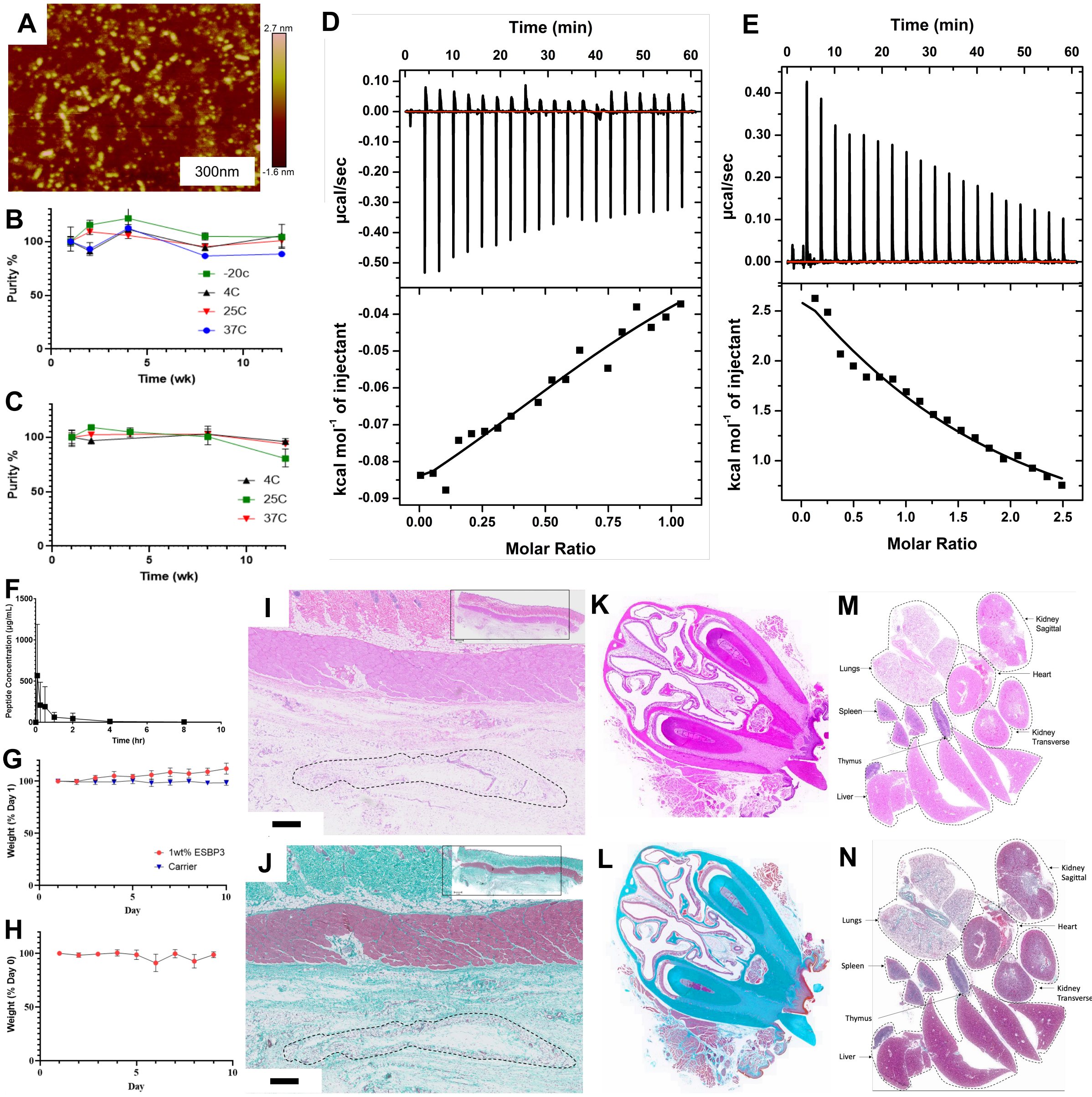

Supplementary Figure 2. Characterization of ESBP3 family of peptides. (A) AFM supported that ESBP3 forms ~ 10-15 nm wide x 1-2 nm thick ribbon-like fibers of µm length that entangle and solvate to create viscous liquids at 0.1-4 w% in saline. **(B)** Lyophilized ESBP3 exhibited exceptional preliminary stability at -20 °C, 4 °C, 25 °C and 37°C over 10 weeks; with LC-MS analysis confirming minimal degradation of ESBP3 over 10 weeks even in aqueous formulation **(C). (D)** Isothermal Titration Calorimetry (ITC) supported self-assembly of ESBP3, and **(E)** binding to spike RBD. **(F)** IV PK of ESBP3 was established by 200µL (10 mg/mL) intravenous bolus injections through the tail vein of Wistar rats (n=8) which showed a concentration peak at 30 min and was eliminated by 8 hour. The observed maximum concentration was ~ 550 µg/mL at 5 min (AUC: 395 µg-hr/mL) following injection. **(G)** IV safety was established by daily repeat dosing with ESBP3 for 10 days causing no significant changes in body weight, animal behavior or adverse events, and gross organ morphological analysis at sacrifice compared to carrier. **(H)**

Subcutaneous bolus (200 µL, 10 mg/mL) administration once with daily blood draws for 10 days demonstrated identical tolerability with no weight loss or adverse event, and no detectable ESBP3 in the plasma. **(I)** H&E and **(J)** Masson's Trichrome (MT) stained sections of Sub-Q implanted boluses at 7 days shows the infiltration of boluses with neutrophils/ macrophages, scale bar 500µm. IN dosing safety was established using 25µL / nostril x 2 daily for 10 days in mice, with nasal turbinate showing no adverse effects as seen in **(K)** H&E and **(L)** MT staining. Imaging whole organs after IN repeat dosing showed no adverse toxicity in heart, lungs, kidneys, liver and thymus or spleen upon histopathologic analysis as seen in **(M)** H&E and **(N)** MT staining.

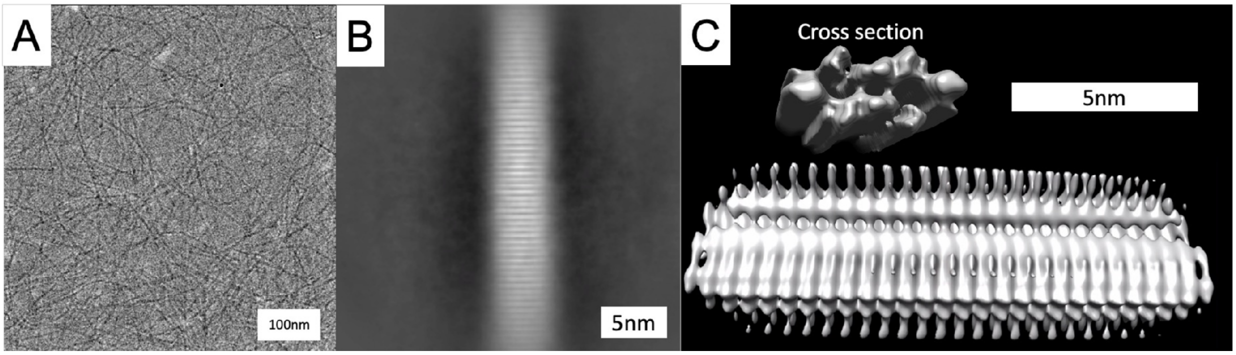

Supplementary Figure 3: Cryo-EM imaging and reconstruction of E1. (A) E1 is the base self-assembling peptide as imaged with cryo-TEM. **(B)** Class averaged structures of 750 micrographs at 4.56 Å resolution show a clear banding pattern, that reconstructed gives a preliminary 3D nanofibrillar structure of E1 **(C)** shown in lateral and cross-sectional views.

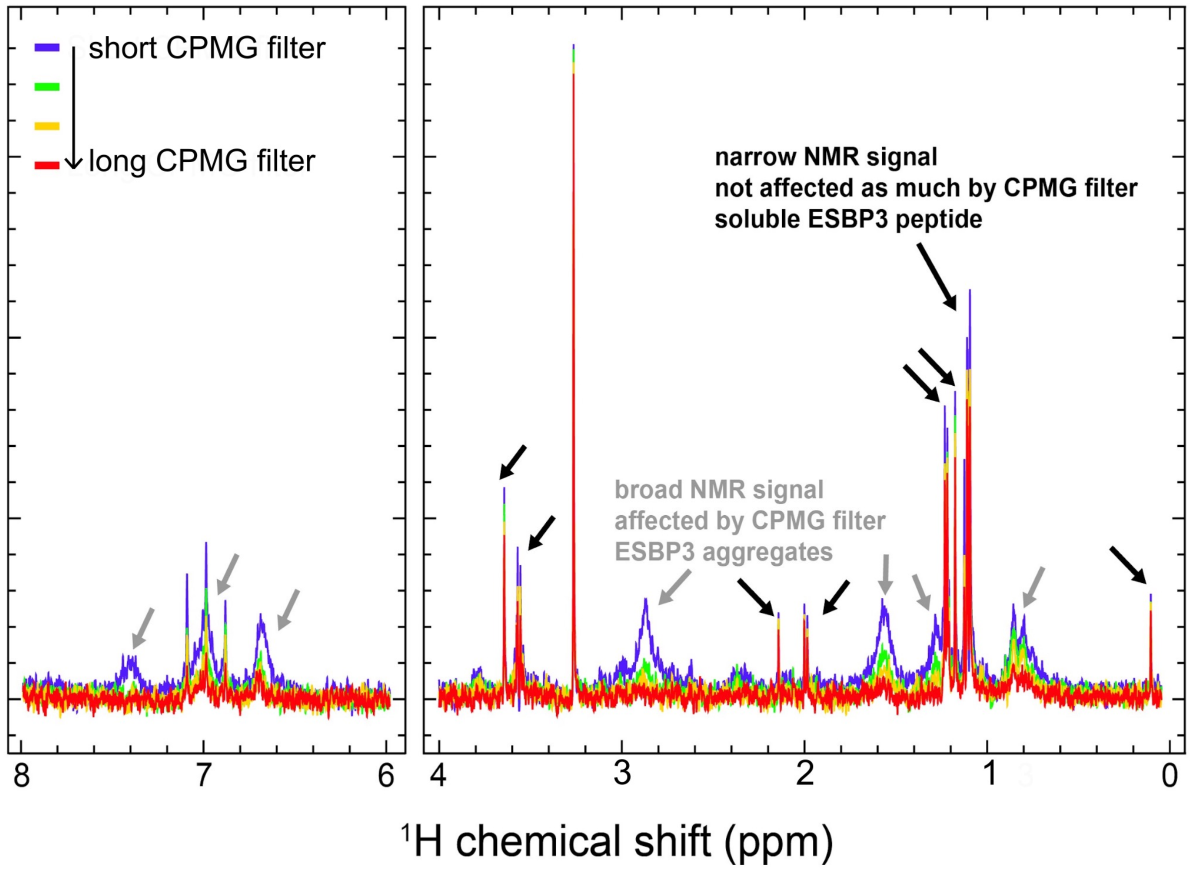

Supplementary Figure 4. Validation of soluble aggregates of ESBP3 using 1D ^{1}H solution NMR CPMG experiments. Overlay of 1D ^{1}H CPMG NMR spectra of ESBP3 peptide (natural isotopic abundance at 1 mg/ml) recorded using a short CPMG relaxation filter (blue), moderate CPMG relaxation filters (green, yellow), and long CPMG relaxation filter (red). Large molecular species with a short T_2 values (i.e. oligomeric species) were filtered out by using a longer CPMG filter, while small molecular species with large T_2 values (i.e. monomeric peptides) were not. Broad NMR peaks (significantly affected by the CPMG filter) correspond to soluble aggregates or oligomers of ESBP3 peptide. The broad NMR signals of the soluble aggregates were filtered out by longer CPMG filters due to their small T_2 relaxation values endowed by the large molecular weight of the aggregates. Narrow NMR peaks (generally unaffected by the CPMG filter) correspond to soluble ESBP3 monomeric peptide in non-aggregated form. The narrow NMR signals were not filtered out due to their large T_2 relaxation value endowed by the low molecular weight of the monomeric peptide state.

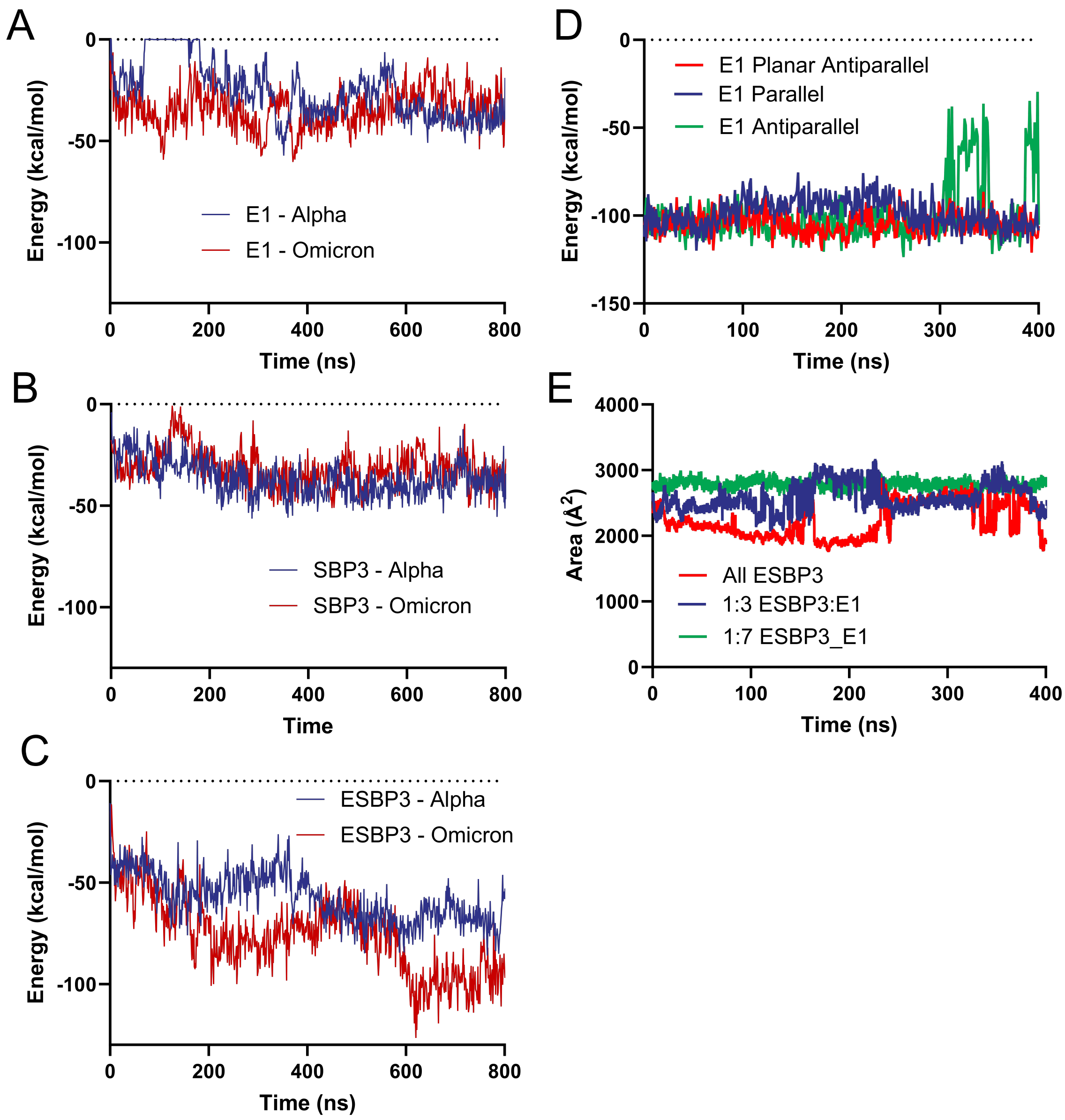

Supplementary Figure 5. Evaluation of monomer binding energy on variants. Simulations for 800 ns for E1 monomer against Alpha and Omicron variants (A), SBP3 monomer against Alpha and Omicron variants (B), and ESBP3 monomer against Alpha and Omicron variants (C), supported that the peptides maintained binding to Spike with comparable coupling energy. (D) Time - energy analysis of the Van der Waals & Electrostatic energy vs. time, used to determine that the system has entered an equilibrated state for MM-GBSA energy. (E) Accessible Surface Area vs. time plots establishing stabilized fibers for analysis of the last 40ns of simulation.

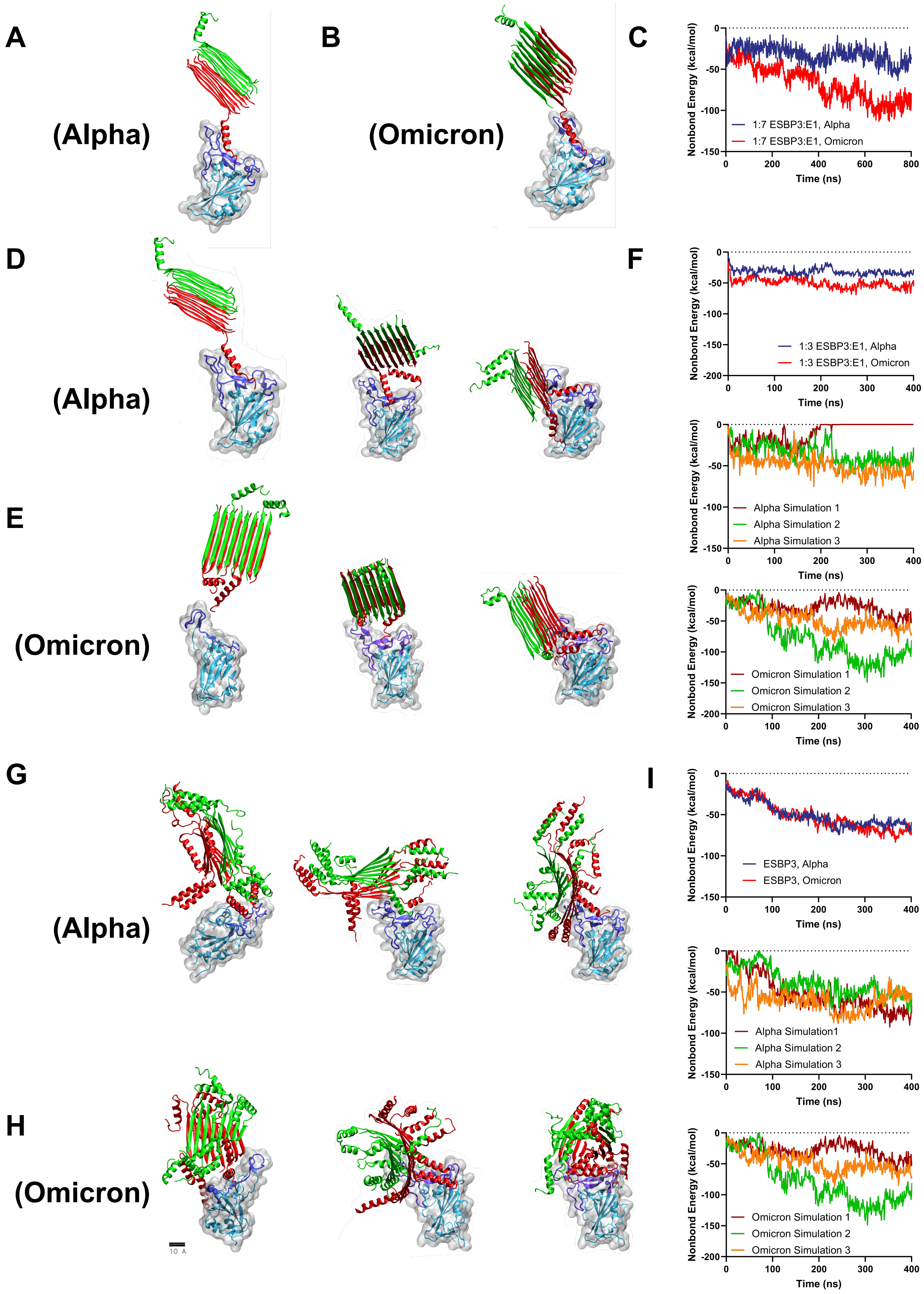

A
(Alpha)
B
(Omicron)
C
Nonbond Energy (kcal/mol)
1:7 ESBP3:E1, Alpha
1:7 ESBP3:E1, Omicron
Time (ns)
D
(Alpha)
F
1:3 ESBP3:E1, Alpha
1:3 ESBP3:E1, Omicron
Alpha Simulation 1
Alpha Simulation 2
Alpha Simulation 3
E
(Omicron)
Omicron Simulation 1
Omicron Simulation 2
Omicron Simulation 3
G
(Alpha)
I
ESBP3, Alpha
ESBP3, Omicron
Alpha Simulation1
Alpha Simulation 2
Alpha Simulation 3
H
(Omicron)
10 A
Omicron Simulation 1
Omicron Simulation 2
Omicron Simulation 3

Supplementary Figure 6. MD Energy Estimates of Fibers on Receptors. (A) Simulation of 1:7 ESBP3:E1 ratios on the Alpha (B.1.1.7) variant for 400 ns showed interactions between the SBP3 bioactive domain and RBD, with some nonspecific interaction with the E1 domains. **(B)** The same nonspecific interactions appeared more pronounced in omicron both qualitatively and quantitatively with larger nonbond and electrostatic interactions **(C). (D)** The same 400 ns simulations were performed in triplicate for 1:3 ESBP3:E1 ratios to sample more of the energy landscape that becomes available when multiple bioactive domains are interacting with the receptor. **(E)** Simulations against omicron show similar nonspecific interactions between E1 and the Spike RBD, we also notice that there is some internal interaction between SBP3 domains on the fiber, consistent with the reduce solvent accessible surface area (Figs. 4D-F). **(F)** Energy plots of Alpha, Omicron, and their average values show an insignificant change in binding energy between alpha and omicron variants, suggesting the ability to minimize “Antibody Escape” by excessive topology changes on the RBD of mutant sequences. **(G)** All ESBP3 fibers showed qualitatively more disorganization and interaction between SBP3 bioactive domains. **(H)** This phenomenon was also observed on Omicron. **(I)** Van der Waals and Electrostatic interactions were the highest for all ESBP3 fibers. (Scale bar for all simulation images is 10Å)
