# Supplementary material for: Antiviral fibrils of self-assembled peptides with tunable compositions
Source: Nat Commun. 2024 Feb 7;15:1142. doi: 10.1038/s41467-024-45193-3 (PMC10850501; doi:10.1038/s41467-024-45193-3)
Supplement: Supplementary file 3 — Description of Additional Supplementary Files [file 41467_2024_45193_MOESM3_ESM.pdf]

Supplementary Movie 1:
Description: Video showing 1:7 ESBP3:E1 peptides in a fiber interacting with Spike from SARS-CoV-2 Omicron variant.

Supplementary Movie 2:
Description: Video showing 1:3 ESBP3:E1 peptides in a fiber interacting with Spike from SARS-CoV-2 Omicron variant.

Supplementary Movie 3:
Description: Video showing the association of all ESBP3 peptides in a fiber interacting with Spike from SARS-CoV-2 Omicron variant.
