# Supplementary figures and images for: Antiviral fibrils of self-assembled peptides with tunable compositions
Source: Nat Commun. 2024 Feb 7;15:1142. doi: 10.1038/s41467-024-45193-3 (PMC10850501; doi:10.1038/s41467-024-45193-3)

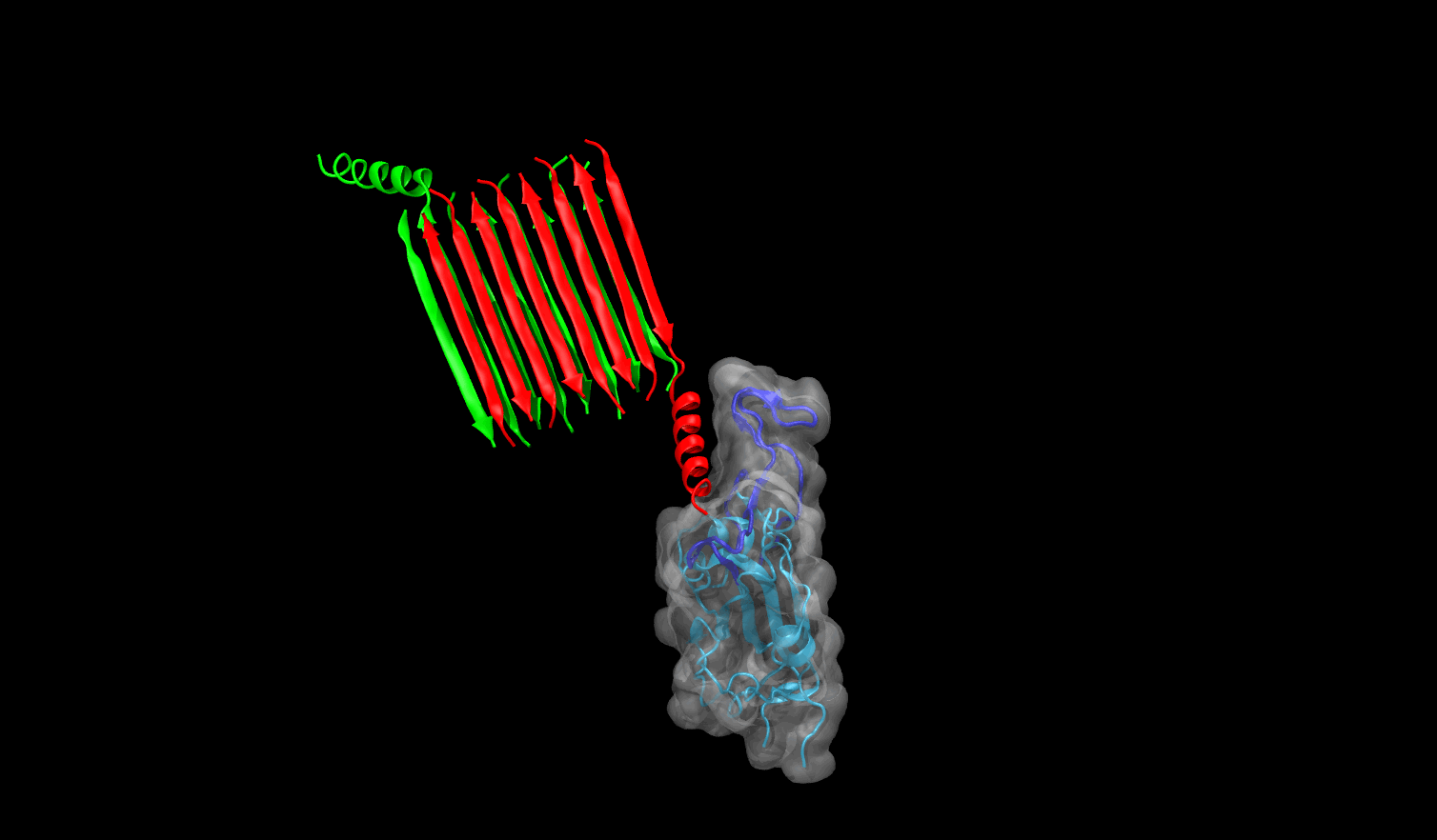

Supplement: Supplementary file 4 — Supplementary Movie 1 [file 41467_2024_45193_MOESM4_ESM.gif]

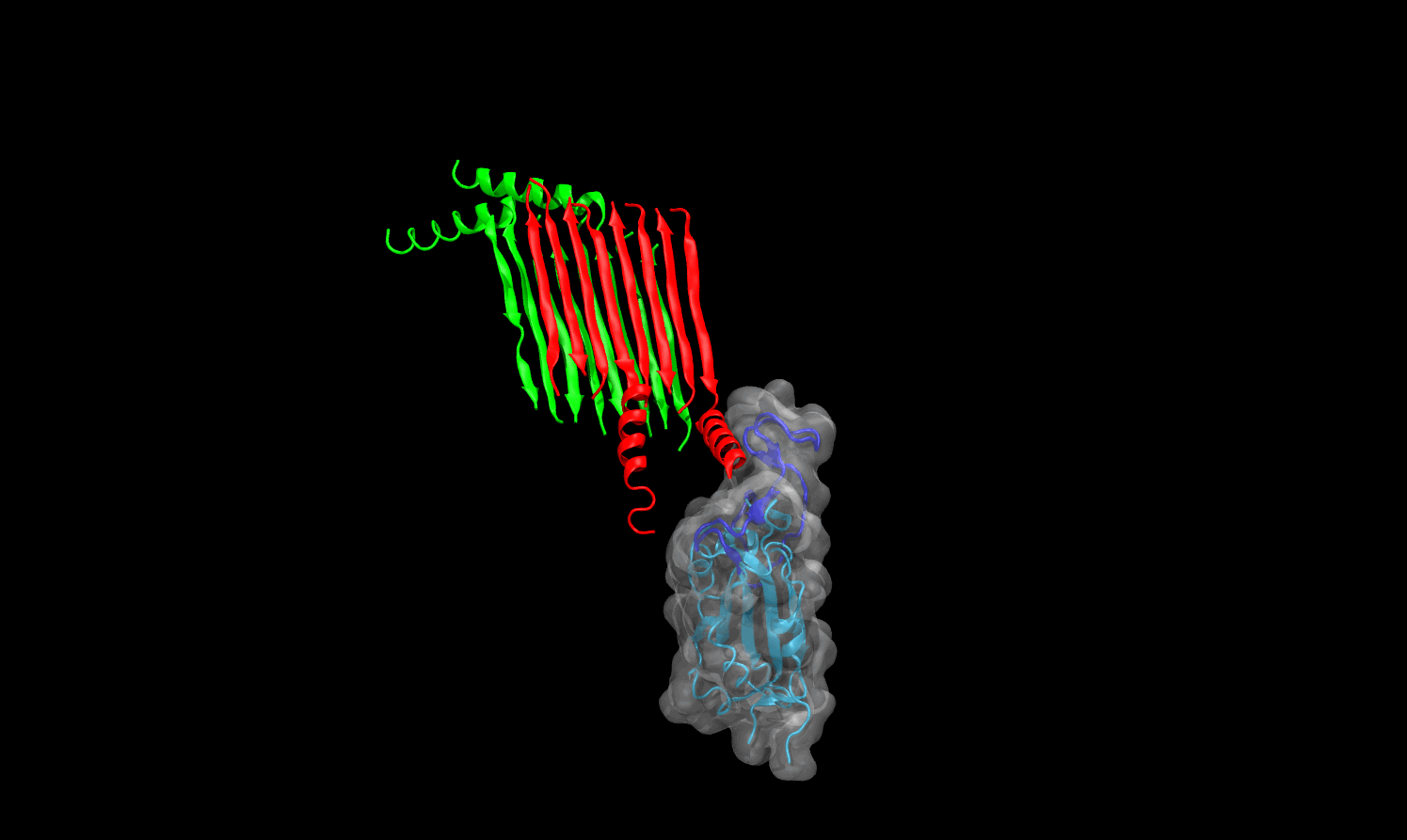

Supplement: Supplementary file 5 — Supplementary Movie 2 [file 41467_2024_45193_MOESM5_ESM.gif]

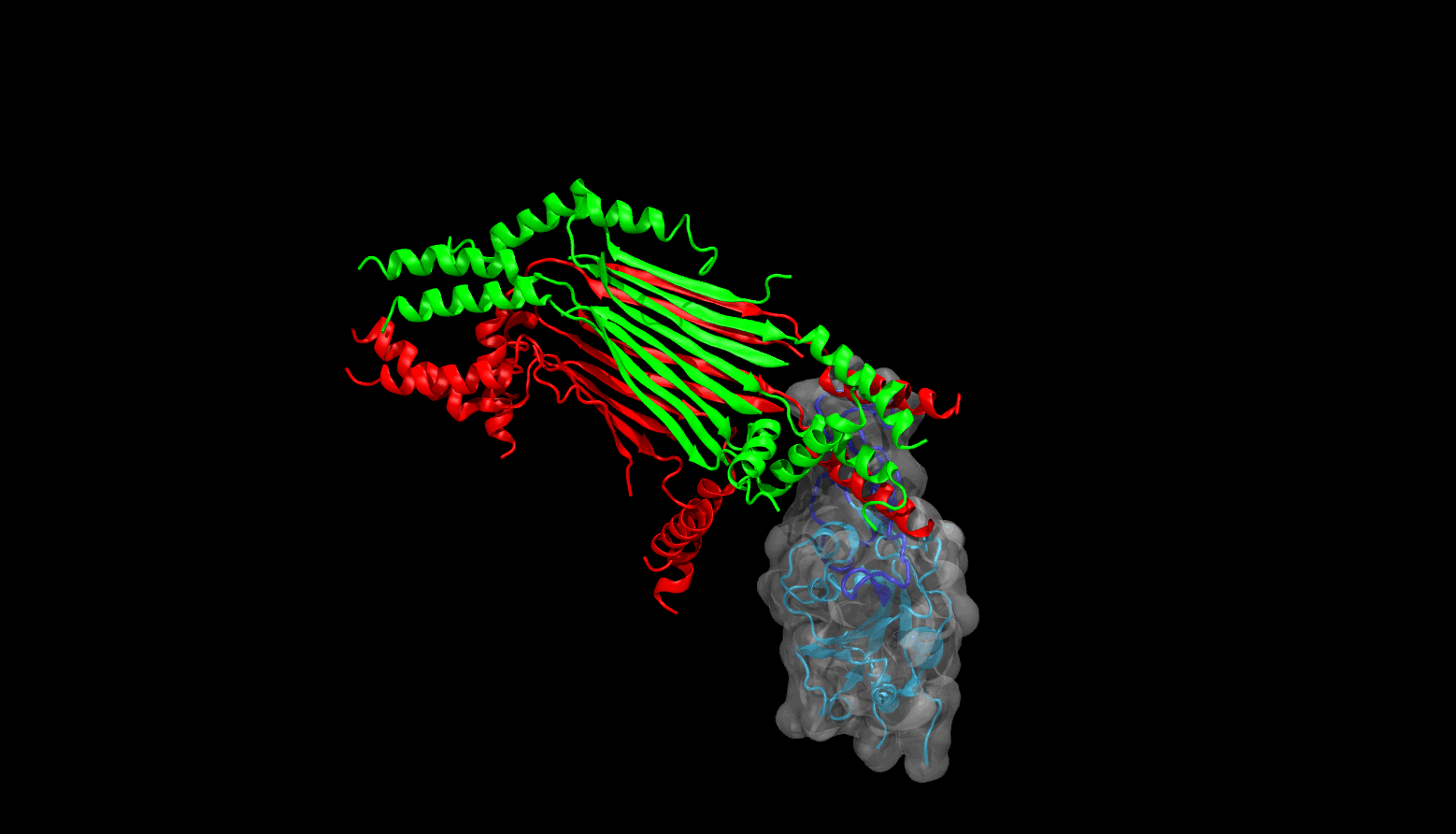

Supplement: Supplementary file 6 — Supplementary Movie 3 [file 41467_2024_45193_MOESM6_ESM.gif]
