# Supplementary material for: Antiviral fibrils of self-assembled peptides with tunable compositions
Source: Nat Commun. 2024 Feb 7;15:1142. doi: 10.1038/s41467-024-45193-3 (PMC10850501; doi:10.1038/s41467-024-45193-3)
Supplement: Supplementary file 7 — Reporting Summary [file 41467_2024_45193_MOESM7_ESM.pdf]

nature portfolio

Corresponding author(s): Petr Kral & Vivek Kumar

Last updated by author(s): Dec 18, 2023

Reporting Summary

Nature Portfolio wishes to improve the reproducibility of the work that we publish. This form provides structure for consistency and transparency in reporting. For further information on Nature Portfolio policies, see our Editorial Policies and the Editorial Policy Checklist.

Statistics

For all statistical analyses, confirm that the following items are present in the figure legend, table legend, main text, or Methods section.

n/a	Confirmed	
☐	☒	The exact sample size (n) for each experimental group/condition, given as a discrete number and unit of measurement
☐	☒	A statement on whether measurements were taken from distinct samples or whether the same sample was measured repeatedly
☐	☒	The statistical test(s) used AND whether they are one- or two-sided<br>*Only common tests should be described solely by name; describe more complex techniques in the Methods section.*
☒	☐	A description of all covariates tested
☒	☐	A description of any assumptions or corrections, such as tests of normality and adjustment for multiple comparisons
☐	☒	A full description of the statistical parameters including central tendency (e.g. means) or other basic estimates (e.g. regression coefficient) AND variation (e.g. standard deviation) or associated estimates of uncertainty (e.g. confidence intervals)
☐	☒	For null hypothesis testing, the test statistic (e.g. F, t, r) with confidence intervals, effect sizes, degrees of freedom and P value noted<br>*Give P values as exact values whenever suitable.*
☒	☐	For Bayesian analysis, information on the choice of priors and Markov chain Monte Carlo settings
☒	☐	For hierarchical and complex designs, identification of the appropriate level for tests and full reporting of outcomes
☒	☐	Estimates of effect sizes (e.g. Cohen's d, Pearson's r), indicating how they were calculated

Our web collection on statistics for biologists contains articles on many of the points above.

Software and code

Policy information about availability of computer code

Data collection	Pymol 2.5.4, Rosetta 4.2, NAMD 2.14, HADDOCK 2.4, BeAtMuSiC 1.0, SWISS-MODEL Expasy web server, PRODIGY, BAlaS.
Data analysis	Statistical data was analyzed with GraphPad 10.1.2 and R 4.2.1.

For manuscripts utilizing custom algorithms or software that are central to the research but not yet described in published literature, software must be made available to editors and reviewers. We strongly encourage code deposition in a community repository (e.g. GitHub). See the Nature Portfolio guidelines for submitting code & software for further information.

Data

Policy information about availability of data

All manuscripts must include a data availability statement. This statement should provide the following information, where applicable:

- Accession codes, unique identifiers, or web links for publicly available datasets
- A description of any restrictions on data availability
- For clinical datasets or third party data, please ensure that the statement adheres to our policy

The authors declare that the data supporting the findings of this study are available within the paper and its Supplementary Information files. Input files for all MD simluations are available: https://github.com/jbdoddo/COVID_PEPTIDE_MS . Should any raw data files be needed in another format they are available from the corresponding author upon reasonable request. Source data are provided with this paper within the "Source Data" file.

Human research participants

Policy information about studies involving human research participants and Sex and Gender in Research.

Reporting on sex and gender	NA
Population characteristics	NA
Recruitment	NA
Ethics oversight	NA

Note that full information on the approval of the study protocol must also be provided in the manuscript.

Field-specific reporting

Please select the one below that is the best fit for your research. If you are not sure, read the appropriate sections before making your selection.

[x] Life sciences   [ ] Behavioural & social sciences   [ ] Ecological, evolutionary & environmental sciences

For a reference copy of the document with all sections, see nature.com/documents/nr-reporting-summary-flat.pdf

Life sciences study design

All studies must disclose on these points even when the disclosure is negative.

Sample size	Sample sizes were initially chosen at n=3/4 for in vitro and in vivo assays, based on the preliminary effect size derived from pilot experiments conducted for all studies, some in vitro test groups sample size was expanded in replicate experiments to determine statistical significance or the lack thereof.
Data exclusions	No data was excluded from analysis.
Replication	Experiments were repeated independently at least 3 times (in vitro efficacy and in vivo safety) with different batches of peptide and at different times. In vivo safety was evaluated in multiple animals, with different delivery routes and different batches of peptide.
Randomization	For in vivo studies, safety of the scaffold was evaluated. The was no need for randomization as only 1 experimental group was evaluated for each construct.
Blinding	Samples for in vitro testing were blinded to avoid bias in pseudovirus and live virus testing. Blinding was not possible in in vivo testing given the nature of the safety studies that used a specific peptide.

Reporting for specific materials, systems and methods

We require information from authors about some types of materials, experimental systems and methods used in many studies. Here, indicate whether each material, system or method listed is relevant to your study. If you are not sure if a list item applies to your research, read the appropriate section before selecting a response.

Materials & experimental systems

n/a	Involved in the study	
[x]	[ ]	Antibodies
[ ]	[x]	Eukaryotic cell lines
[x]	[ ]	Palaeontology and archaeology
[ ]	[x]	Animals and other organisms
[x]	[ ]	Clinical data
[x]	[ ]	Dual use research of concern

Methods

n/a	Involved in the study	
[x]	[ ]	ChIP-seq
[x]	[ ]	Flow cytometry
[x]	[ ]	MRI-based neuroimaging

Eukaryotic cell lines

Policy information about cell lines and Sex and Gender in Research

Cell line source(s)	All cells and cells lines were acquired from ATCC. HEK-293 (ATCC CRL-1573) and Vero E6 (ATCC CRL-1586) cells were used.
Authentication	Cell lines were used within the first 2-3 passages from the supplier (ATCC) as per their OA/QC and recommended protocols. Cells were not otherwise authenticated.
Mycoplasma contamination	Cells were not tested for mycoplasm contamination.
Commonly misidentified lines (See ICLAC register)	No commonly misidentified cell lines were used in the study.

Animals and other research organisms

Policy information about studies involving animals; ARRIVE guidelines recommended for reporting animal research, and Sex and Gender in Research

Laboratory animals	All animals used were laboratory strain (C57BL/6 mice 15-20g, 8-12 weeks old; Wistar rats 200-250g, 8-12 weeks old). Housing conditions for the mice/rats, have 12 hr dark/light cycle, ambient temperature, and humidity.
Wild animals	No field collected samples were used in the study.
Reporting on sex	Sex was not considered in the study design, only female animals were used.
Field-collected samples	No field collected samples were used in the study.
Ethics oversight	All animal protocols were approved by the NJIT-Rutgers IACUC.

Note that full information on the approval of the study protocol must also be provided in the manuscript.
